# Uncovering the molecular pathogenesis of congenital hyperinsulinism by panel gene sequencing in 32 Chinese patients

**DOI:** 10.1002/mgg3.162

**Published:** 2015-06-29

**Authors:** Zi‐chuan Fan, Jin‐wen Ni, Lin Yang, Li‐yuan Hu, Si‐min Ma, Mei Mei, Bi‐jun Sun, Hui‐jun Wang, Wen‐hao Zhou

**Affiliations:** ^1^ Department of NeonatologyChildren's Hospital of Fudan UniversityShanghaiChina; ^2^ Key Laboratory of Birth DefectChildren's Hospital of Fudan UniversityShanghaiChina; ^3^ Key Laboratory of Neonatal DiseasesMinistry of HealthChildren's HospitalFudan UniversityShanghaiChina

**Keywords:** Clinical diagnosis, congenital hyperinsulinism, mutation spectra

## Abstract

Congenital hyperinsulinism (CHI) has been mostly associated with mutations in seven major genes. We retrospectively reviewed a cohort of 32 patients with CHI. Extensive mutational analysis (*ABCC8*,*KCNJ11*,*GCK*,*GLUD1*,*HADH*,*HNF4A,* and *UCP2*) was performed on Ion torrent platform, which could analyze hundreds of genes simultaneously with ultrahigh‐multiplex PCR using up to 6144 primer pairs in a single primer pool and address time‐sensitive samples with single‐day assays, from samples to annotated variants, to identify the genetic etiology of this disease. Thirty‐seven sequence changes were identified, including in *ABCC8*/*KCNJ11* (*n* = 25, 65.7%), *GCK* (*n* = 2), *HNF4A* (*n* = 3), *GLUD1* (*n* = 2), *HADH* (*n* = 4), and *UCP2* (*n* = 1); these mutations included 14 disease‐causing mutations, eight rare SNPs, 14 common SNPs, and one novel mutation. Mutations were identified in 21 of 32 patients (65.6%). Among the patients with an identified mutation, 14 had mutations in *ABCC8*, one of which was combined with a *GLUD1* mutation. Four patients had mutations in *KCNJ11*, 1 had a *GCK* mutation, 1 had a mutation in *HADH*, and two had a mutation in *HNF4A*. Among the 32 patients, the age at the onset of hyperinsulinemia ranged from the neonatal period to 1 year of age; five patients underwent a pancreatectomy due to intractable hyperinsulinemia. This study describes novel and previously identified mutations in patients with CHI. The spectrum of mutations in CHI patients represents an important tool for the diagnosis and prognosis of CHI patients in the Chinese population as well as for the genetic counseling of CHI families.

## Introduction

Congenital hyperinsulinism (CHI) is a rare disease of hypoglycemia due to deregulated and excessive insulin secretion, with an estimated incidence of 1/50000 for severe cases and a high incidence of 1/2675 in consanguineous populations (Mathew et al. 1988; Bruining 1990). CHI is heterogeneous with respect to its clinical presentation, genetics, and response to treatment (Sempoux et al. 1998; de Lonlay et al. 2002; Meissner and Mayatepek 2002; Flanagan et al. 2011a). Clinical manifestations of CHI, which occur predominantly in neonates and infants, include large birth weight for gestational age, seizures, cyanosis, and coma (Hussain and Aynsley‐Green 2000). The severity of the disease varies from a mild form, which responds to medical treatment, to a severe drug‐resistant form, which may require resection of the pancreas. The most common causes of CHI are mutations in *KCNJ11* (OMIM #600937) and *ABCC8* (OMIM #600509), which encode the two subunits of the adenosine triphosphate‐sensitive potassium channels (ATP‐sensitive K‐ATP channels) in pancreatic *β*‐cells (Thomas et al. 1995, 1996; Flanagan et al. 2009). Recessive mutations in these genes cause hyperinsulinism that is unresponsive to treatment with channel agonists, such as diazoxide, whereas dominant mutations have been associated with diazoxide‐responsive disease. Other less frequent causes of CHI include mutations in the following genes: *GLUD1* (OMIM #138130), *GCK* (OMIM #138079), *HADH* (OMIM #601609), SLC16A1 (OMIM# 600682), *HNF4A* (OMIM# 600281), and *UCP2* (OMIM# 607447); these proteins are involved in different metabolic pathways and lead to alterations in insulin secretion (Gonzalez‐Barroso et al. 2008; James et al. 2009). Most patients with *GLUD1*,* HADH*, and *HNF4A* mutations exhibit a good response to diazoxide. Heterozygous activating *GCK* mutations cause CHI, which in most cases is medically responsive, but in some cases, surgery may be required (Christesen et al. 2008; Sayed et al. 2009). Identification of a *GLUD1* or *HADH* mutation informs clinicians of the protein‐sensitive nature of the hypoglycemia, allowing for dietary manipulation (protein restriction) as a useful, and sometimes mandatory, adjunct to diazoxide therapy in controlling recurrent hypoglycemic episodes (Bahi‐Buisson et al. 2008; Flanagan et al. 2011b). Furthermore, the identification of an *HNF4A* mutation in a proband would identify infants who are at a risk of developing maturity‐onset diabetes of the young (Yamagata et al. 1996; Kapoor et al. 2008). Unless the mutation has arisen de novo, one of the parents, and potentially other family members, will be heterozygous for the *HNF4A* mutation and will be at an increased risk of developing diabetes, if it has not already developed (Flanagan et al. 2010). For these patients, a genetic diagnosis is important because this monogenic form of diabetes can be successfully managed with low‐dose sulfonylureas.

In this article, we identified the genetic characteristics of 32 Chinese CHI patients; this characterization will lead to improved strategies for genetic screening and prenatal diagnosis. Genetic analyses can also help in the treatment of CHI and in genetic counseling for this condition.

## Materials and Methods

### Subjects and DNA extraction

We retrospectively reviewed a cohort of 32 patients with CHI from October 2009 to June 2014. The samples used in this study were collected with the approval of the ethics committees of Children's Hospital, Fudan University, and the study was conducted according to the principles of the Declaration of Helsinki. It is difficult to set definitive diagnostic criteria for CHI. Several authors have proposed different standards for diagnosing CHI (Hussain 2008; Arnoux et al. 2011; Mohamed et al. 2012; Petraitiene et al. 2014). A diagnosis of CHI in this study was made if serum insulin was detectable (>2 mU/L) concurrent with hypoglycemia (blood glucose <2.6 mmol/L) along with evidence of elevated glucose requirements (glucose infusion rate >8 mg/kg/min). Patients with intrauterine growth retardation, asphyxia at birth, or congenital malformations were excluded.

Genomic DNA was extracted from whole blood using the QIAamp DNA Blood Mini Kit (Qiagen, Hilden, Germany). The DNA concentration was measured using a Nano‐Drop Spectrophotometer (ND‐1000; Thermo Fisher Scientific, Waltham, MA).

### Panel gene sequencing

The analysis of the protein‐coding regions of the human *ABCC8* (NM_000352.4), *KCNJ11* (NM_000525.3), *GCK* (NM_000162.3), *GLUD1* (NM_005271.3), *HADH* (NM_001184705.2), *HNF4A* (NM_000457.4) and *UCP2* (NM_003355.2) genes was performed using Ion Torrent PGM^™^. The complete target gene set included 65 amplicons (from 222 bp to 6597 bp) covering all the exons and at least 20 bp of all the intron/splice sites. The library was prepared according to the instructions provided by the manufacturers of the kits for fragmentation (Ion Shear, Life Technologies, Grand Island, NY, USA) and adapter and barcode ligation (Ion Xpress Barcode Adapter Kit, Life Technologies). We used the Ion OneTouch^™^ system (Life Technologies) to clonally amplify pooled barcoded libraries on Ion Sphere^™^ particles (Life Technologies, Grand Island, NY, USA). Torrent Suite^™^ software (Life Technologies, Grand Island, NY, USA) was used to compare the base calls. NextGene^™^ software (Aurora, Colorado, USA) was used to read the alignments and to call the variants based on the human genomic reference hg19 (NCBI). The variants selected for further analysis met the following criteria: (1) the variant was detected in sequence reads for both strands, (2) a minimum coverage of 10× was achieved, (3) the variant reads represented >20% of the sequence reads at a particular site, and (4) the targeted region covered all the exons and at least 20 bp of all the intron/splice sites.

### Variant analysis

To evaluate all the filtered variants, the Human Gene Mutation Database (http://www.hgmd.cf.ac.uk/ac/index.php), Online Mendelian Inheritance in Man Database (http://www.ncbi.nlm.nih.gov/omim), Single‐Nucleotide Polymorphism Database (dbSNP 137, http://www.ncbi.nlm.nih.gov/snp), and 1000 Genomes Project (http://www.1000genomes.org/) were consulted. Furthermore, to test the pathogenicity of all the variants, the Polymorphism Phenotyping (PolyPhen‐2; http://genetics.bwh.harvard.edu/pph2), [Ramensky et al. 2002; Sunyaev et al. 2001). Sorting Intolerant From Tolerant (SIFT; http://sift.jcvi.org) (Hon et al. 2009) and Mutation Taster (http://www.mutationtaster.org/) (Schwarz et al. 2014) pathogenicity prediction web tools were used. The web tool ESEfinder (http://rulai.cshl.edu/tools/ESE/) was used to detect alterations in exonic splicing enhancers (ESE) due to nucleotide changes. Changes affecting highly conserved positions were generally interpreted as having damaging effects. Related variants were assessed in 50 normal controls.

### Validation by Sanger sequencing

The variants were validated by PCR followed by direct Sanger sequencing using an automated sequencer (ABI 3130 Genetic Analyzer; Applied Biosystems, Foster City, CA).

## Results

### Clinical evaluation

The clinical features of the 32 CHI patients (15 males and 17 females) are summarized in Table 1. The average birth weight was 3708 g. Overall, 20 of the 32 patients were large for gestational age (>90th percentile), confirming that CHI patients tend to have a high birth weight (Grimberg et al. 2001). Twenty‐six patients experienced neonatal disease onset. Most of the patients required a high glucose infusion rate to maintain euglycemia. Sixteen patients showed varying degrees of convulsions, highlighting the high risk of cerebral injury. Five patients underwent a pancreatectomy because of uncontrolled hypoglycemia. We have successfully followed two families.

**Table 1:** Clinical features of Chinese CHI patients

Patient	Sex	Birth weight (g)	Gestational age (weeks)	LGA (>90 percentile)	Age at onset of symptoms (days)	Blood glucose levels at presentation (mg/dl)	Blood Insulin (uIU/mL)	Glucose infusion rate (mg/kg/min)	Diazoxide treatment/ Response	Pancreatectomy/ Histology	Convulsion
1^a^^,^ d	M	4250	39	Y	Neonatal	1.3	26.7	12.5	Y/N	Y/Focal	Y
2^a^	M	4500	41	Y	Neonatal	1.8	13.7	15	–	–	N
3^a^	F	5400	38 + 3	Y	Neonatal	0.7	10.5	12	–	N	Y
4^a^	F	3450	39 + 6	N	Infancy	1.6	16.3	12.5	–	Y/Focal	Y
5^a^	F	4300	39 + 6	Y	Neonatal	2.4	10.8	12.5	–	–	N
6^a^	F	3900	40	Y	Neonatal	1.3	28.9	11	–	–	N
7^a^	M	3300	37 + 5	N	Infancy	1.4	12.83	10	–	–	N
8^a^	M	3200	38	N	Neonatal	1.9	12.9	12.5	–	–	N
9^a^	M	5700	39 + 2	Y	Neonatal	0.9	39.1	10	–	–	Y
10^a^^,^ f	F	3950	39 + 1	Y	Neonatal	1.7	29.6	15	–	–	N
11^a^	M	3750	37 + 3	Y	Neonatal	1.2	2.4	12.5	–	–	Y
12^a^	F	3600	38	Y	Neonatal	1.8	18.5	12.5	–	–	N
13^a^	M	3900	39 + 2	Y	Neonatal	0.57	22.4	12.5	Y/N	Y/Focal	Y
14^b^	M	4415	35	Y	Neonatal	1.5	61.6	10	Y/Y	N	Y
15^b^	F	4400	43	Y	Infancy	1.5	14.28	12.5	–	N	Y
16^a^^,^ b	F	4150	38 + 6	Y	Neonatal	1.6	88.1	12.5	–	–	Y
17^b^	F	3950	40	Y	Neonatal	2.1	12.7	10	–	–	Y
18^c^	M	2000	38 + 5	N	Neonatal	1.8	4.3	10	–	N	Y
19^d^	F	3700	39	Y	Neonatal	2.0	17.5	12	–	–	Y
20^e^	F	3350	38 + 4	N	Infancy	1.0	8.3	10	Y/N	Y/Focal	N
21^e^	F	4000	39 + 3	Y	Neonatal	1.2	11.1	10	Y/N	Y/Diffuse	Y
22	M	1950	37 + 5	N	Neonatal	1.3	15.3	12.5	–	N	N
23	M	3350	39 + 4	N	Neonatal	1.9	12.7	10	–	N	N
24	M	4550	41	Y	Neonatal	1.7	13.6	10	–	N	Y
25	F	3150	38	N	>1 year	2.2	6.6	10	–	N	N
26	F	4800	39 + 4	Y	Neonatal	1.2	220	15	–	–	Y
27	M	2650	39 + 2	N	Neonatal	1.8	12.2	10	–	–	N
28	F	1905	38 + 3	N	Neonatal	2.1	8.6	10	Y/N	–	N
29	F	3850	39	Y	Infancy	2.1	3.47	10	–	–	Y
30	M	3000	38	N	Neonatal	2.4	15.8	12.5	–	–	N
31	M	2550	38 + 1	N	Neonatal	1.9	3.3	10	–	–	N
32	F	3750	39	Y	Neonatal	1.6	11.7	10	–	–	N

a Patients with ABCC8 gene variants.

b Patients with KCNJ11 gene variants.

c Patients with GCK gene variants.

d Patients with HADH gene variants.

e Patients with HNF4A gene variants.

f Patients with GLUD1 gene variants.

### Sequence changes in CHI‐related genes

A total of 37 nucleotide sequence changes within the within the CHI‐related genes were identified in 21 of 32 (65.6%) patients. Most of which involved the *ABCC8* and *KCNJ11* genes (25/37, 67.6%). The other sequence changes were in *GCK* (*n* = 2), *HNF4A* (*n* = 3), *GLUD1* (*n* = 2), *HADH* (*n* = 4), and *UCP2* (*n* = 1). Nineteen of these sequence changes are classified as potential disease‐causing mutations (Table 2) and the remainder as putative polymorphisms (Table 3).

**Table 2:** Sequence changes found in Chinese CHI patients

Patient	Exon or intron	Mutation at cDNA level	Mutation at protein level	Mutation type	Prediction (polyphen2/ SIFT/Mutation Taster)	SNP/MAF	HGMD ID	Conservation	Control
ABCC8 (NM_000352.4)									
1	E 21	c.2506C>T	p.R836*	Nonsense	Disease causing (‐/damaging/damaging)	rs72559722/NA	CM001605	–	0/50
	E 23	c.2797C>T	p.R933*	Nonsense	Disease causing (‐/damaging/damaging)	–	CM060771	–	0/50
2	E 23	c.2797C>T	p.R933*	Nonsense	Disease causing (‐/damaging/damaging)	–	CM060771	–	0/50
3	E 37	c.4516G>A	p.E1506*	Missense	Disease causing (‐/damaging/damaging)	rs137852671/ NA	CM011262	highly	0/50
4	I 22	c.2557‐1G>C	Unknown	Aberrant splicing	Disease causing (‐/damaging/damaging)	–	–	–	0/50
5	E 37	c.4516G>A	p.E1506*	Missense	Disease causing (‐/damaging/damaging)	rs137852671/ NA	CM011262	Highly	0/50
6	E 25	c.3124_3126delACCinsCAGCCAGGAACTG	p.T1042Qfs*75	Frameshift	Disease causing (‐/damaging/damaging)	–	–	–	0/50
7	E 25	c.3124_3126delACCinsCAGCCAGGAACTG	p.T1042Qfs*75	Frameshift	Disease causing (‐/damaging/damaging)	–	–	–	0/50
8	E 39	c.4697_*+5del	Unknown	Deletion	Unknown (‐/‐/‐)	–	–	–	0/50
9	I 8	c.1176+1G>A	Unknown	Aberrant splicing	Disease causing (‐/damaging/damaging)	–	–	–	0/50
	E 21	c.2506C>T	p.R836*	Nonsense	Disease causing (‐/damaging/damaging)	rs72559722/ NA	CM001605	–	0/50
10	E 1	c.80T>C	p.F27S	Missense	Disease causing (possibly damaging/damaging/damaging)	–	CM050964	Highly	0/50
11	E 2	c.220C>T	p.R74W	Missense	Disease causing (possibly damaging/damaging/damaging)	rs201682634/ A = 0.000/1	CM050965	Highly	0/50
12	E 30	c.3748C>T	p.R1250*	Nonsense	Disease causing (‐/damaging/damaging)	–	CM060775	–	0/50
13	E 25	c.2990G>A	p.W997*	Nonsense	Disease causing (‐/damaging/damaging)	–	–	–	0/50
16	E24	c.2857delC	p.Q953Rfs*89	Frameshift	Disease causing (‐/damaging/damaging)	–	–	–	0/50
	E35	c.4284delC	p.V1429Sfs*31	Frameshift	Disease causing (‐/damaging/damaging)	–	–	–	0/50
KCNJ11 (NM_000525.3)									
14	E 1	c.146T>A	p.I49N	Missense	Disease causing (possibly damaging/damaging/damaging)	–	–	Highly	0/50
15	E 1	c.305_306insG	p.S103Pfs*28	Frameshift	Disease causing (‐/damaging/damaging)	–	–	–	0/50
16	E 1	c.843C>T	p.L281L	Synonymous	Disease causing (‐/damaging/damaging)	rs116392938/ A = 0.002/10	–	–	0/50
17	E 1	c.305_306insG	p.S103Pfs*28	Frameshift	Disease causing (‐/damaging/damaging)	–	–	–	0/50
GCK (NM_000162.3)									
18	E 1	c.37_39delAAG	p.K13del	Deletion	Polymorphism (‐/toleratd/ploymorphism)	–	–	–	0/50
HADH (NM_001184705.2)									
1	E 7	c.719G>T	p.T240M	Missense	Polymorphism (benign/damaging/damaging)	rs79116599/ T = 0.004/21	–	Lowly	0/50
19	E 1	c.29G>C	p.R10P	Missense	Disease causing (possibly damaging /damaging/damaging)	–	–	Highly	0/50
	E 1	c.89T>A	p.V30E	Missense	Disease causing (possibly damaging/damaging/damaging)	–	–	Highly	0/50
HNF4A (NM_000457.4)									
20	UTR‐3	c.*7G>A	Unknown	Unknown	Polymorphism (‐/tolerated/ploymorphism)	rs186057842/ A = 0.0005/1	–	–	0/50
21	E 4	c.416C>T	p.T139I	Missense	Disease causing (benign/damaging/damaging)	rs1800961/T = 0.024/120	CM004479	Highly	0/50
GLUD1 (NM_005271.3)									
10	I 9	c.1279‐4A>G	Unknown	Aberrant splicing	Polymorphism (‐/tolerated/ploymorphism)	rs201376212/ C = 0.000/1	–	–	0/50

**Table 3:** Polymorphisms found in the Chinese patients

Mutation at cDNA level	Mutation at protein level	Exon or intron	Mutation type	SNP/MAF	Patients	Control
ABCC8 (NM_000352.4)						
c.207A>G	p.P69P	E 2	Synonymous	rs1048099/G = 0.444/967	7/32	33/50
c.1947C>T	p.K649K	E 14	Synonymous	rs1799858/T = 0.159/347	3/32	13/50
c.1686C>T	p.H562H	E 12	Synonymous	rs1799857/A = 0.385/838	4/32	24/50
c.2117‐3C>T	Unknown	I 15	Aberrant splicing	rs1799854/A = 0.416/907	13/32	45/50
c.3819C>T	p.R1273R	E 31	Synonymous	rs1799859/T = 0.331/720	2/32	13/50
c.4108C>A	p.A1369S	E 33	Missense	rs757110/C = 0.286/622	10/32	45/50
KCNJ11 (NM_000525.3)						
c.67T>C	p.K23E	E 1	Missense	rs5219/T = 0.274/597	7/32	45/50
c.570G>A	p.A190A	E 1	Synonymous	rs5218/A = 0.276/60	5/32	34/50
c.1009C>T	p.V337I	E 1	Missense	rs5215/C = 0.280/610	8/32	45/50
GCK (NM_000162.3)						
c.1256+8G>A	Unknown	I 9	Aberrant splicing	rs2908274/A = 0.376/820	10/32	32/50
GLUD1 (NM_005271.3)						
c.942A>G	p.L314L	E 7	Synonymous	rs9421572/C = 0.216/471	11/32	38/50
HADH (NM_001184705.2)						
c.257T>C	p.L86P	E 2	Missense	rs4956145/T = 0.107/234	29/32	50/50
HNF4A (NM_000457.4)						
c.116‐5C>T	Unknown	I 1	Aberrant splicing	rs745975/T = 0.190/413	7/32	17/50
UCP2 (NM_003355.2)						
c.164G>A	p.A55V	E 4	Missense	rs660339/A = 0.432/940	20/32	40/50

#### ABCC8 gene disease‐causing mutations

**Figure 1:**
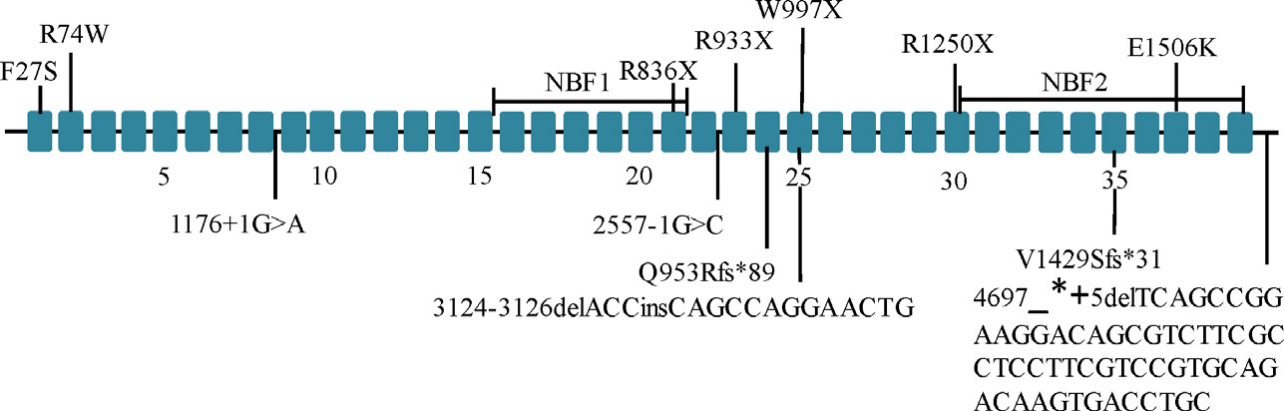
Schematic diagram of the locations of 13 mutations identified in the ABCC8 gene in HI patients. All 39 exons of the ABCC8 gene are depicted as closed boxes and are not drawn to scale. Splice‐site mutations, insertions, and deletions are shown below the gene and missense and nonsense mutations are indicated above the gene.

Thirteen *ABCC8* mutations were identified in 14 of the 32 patients (43.8%) (Table 2 and Fig. 1). Of these, seven mutations have been previously reported, and six were novel. These mutations were not detected in the control group. Of the six novel mutations, two were aberrant splicing mutations, two frameshift, one nonsense, and one deletion.

**Figure 2:**
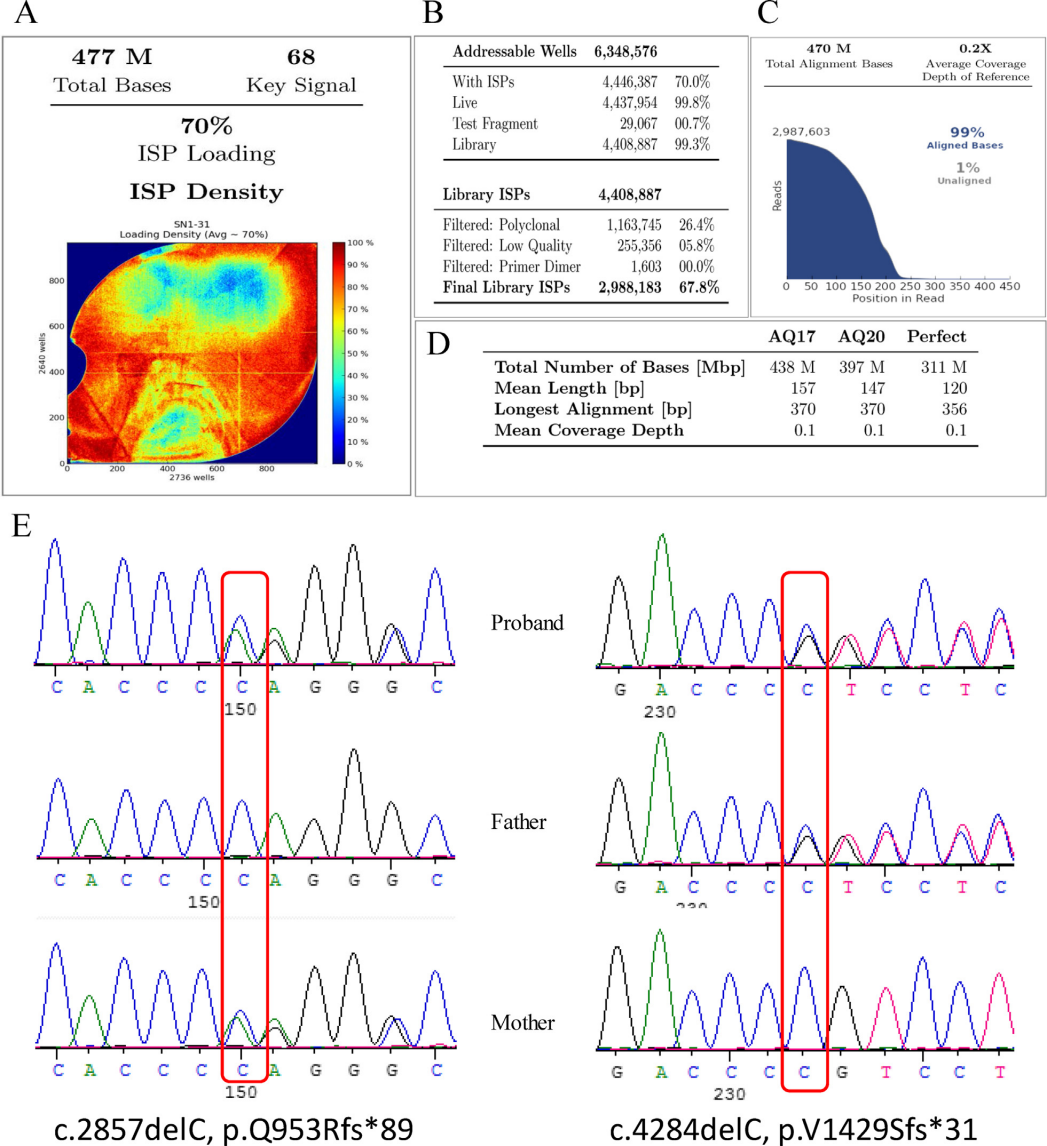
(A) shown the total amount of data is 477 M, (B) the chip achieved 70.0% utilization rate, effective ISPs is 99.8%; (C) shows 99% reads can be compared with the reference sequence, (D) shows the quality inspection, the AQ17 amount of data is 438Mbp and the AQ20 amount of data is 397Mbp. (E) shows the Sanger sequencing results confirmed the compound heterozygous mutation in *ABCC8*, which was inherited from the proband's father and mother, respectively.

One child (patient no. 1) was a compound heterozygote with two reported nonsense mutations in *ABCC8* and a missense mutation in *HADH*, namely p.R836*, p.R933*, and p.T240M; another (patient no. 9) was a compound heterozygote with two mutations in *ABCC8* (c.1176+1G>A and p.R836*). Patient 10 had one heterozygous mutation in *ABCC8* and another in *GLUD1*. The serum ammonia concentration of this patient was 147 *μ*mol/L, which is three times higher than the normal serum ammonia concentration. Patient 16 was a compound heterozygote with two single base deletion variants, which were inherited from his father and mother respectively. (Fig. 2)

#### KCNJ11 gene variant analysis

Four *KCNJ11* mutations were identified in 4 of the 32 patients (12.5%). All of these mutations were novel and were not detected in the control group.

#### HADH gene variant analysis

As shown in Table 2, three mutations were identified in 2 of the 32 patients (6.25%). All of these mutations were novel and were not detected in the control group. One child (patient no. 19) was a compound heterozygote with two missense mutations at highly conserved sites. A missense mutation at a poorly conserved site was detected in patient 1.

#### HNF4A gene variant analysis

Two mutations were identified in 2 of the 32 patients (6.25%); one was a previously reported missense mutation, and the other was a novel mutation in a noncoding region.

#### GCK gene variant analysis

Patient 20 had a 3‐base deletion in *GCK*, which was predicted to be a polymorphism.

## Discussion

In this study, we report the molecular and clinical characterization of 32 Chinese infants with CHI. We found that 10 patients (1, 2, 3, 5, 9, 10, 11, 12, 13, 21) with reported pathogenic mutations in CHI‐related genes were large for gestational age (>90th percentile); only 2 patients (4, 8) with novel and predicted disease‐causing mutations were not LGA, indicating fetal hyperinsulinemia. The analysis of the age at onset of hypoglycemia showed that mutations in CHI‐related genes are more prevalent among patients with early onset disease (<5 days), which is supposedly associated with a worse phenotype. Five patients (1, 4, 13, 20, 21) underwent a pancreatectomy for uncontrolled hypoglycemia, and the families of two patients (27, 28) refused the surgery.

Potential disease‐causing mutations in *ABCC8*/*KCNJ11* were detected in 14 patients, and these mutations are summarized in Table 2. Two recent large‐scale studies [Kapoor et al. 2013). (Snider et al. 2013) reported the identification of *ABCC8*/*KCNJ11* mutation rates of approximately 36.3% (109/300) and 69% (288/417). Likewise, similar studies conducted in Japan (Yorifuji et al. 2011) and Korea (Park et al. 2011) identified *ABCC8*/*KCNJ11* mutation rates in CHI patients of 52.8% (19/36) and 82% (14/17), respectively. Compared to these previous studies, we found mutations in a lower percentage (43.8%) of our patients. Our findings also differed from a recent Chinese study. They found that in a total of 30 CHI patients, the *ABCC8*/*KCNJ11* mutation rate was 37% (11/30). The inconsistency in the *ABCC8*/*KCNJ11* mutation rate in CHI patients between Asian and Western populations may stem from racial differences. The inconsistent inclusion criteria for CHI studies and the small sample size may also contribute to the discrepancies. Large‐scale studies should be conducted to further clarify these inconsistencies.

Compound heterozygous mutations in the *ABCC8* gene have been reported in CHI patients (Sandal et al. 2009; Faletra et al. 2013). In agreement with these studies, our analysis identified three patients (1, 9, 16) who harbored compound heterozygous mutations (1: p.R836* and p.R933*; 9: c.1176+1G>A and p.R836*; 16: p.Q953Rfs*89 and p.V1429Sfs*31) in *ABCC8*. The p.R836* and p.R933* mutations introduce a stop signal that abruptly terminates protein synthesis, resulting in a truncated protein product. The p.R836X mutation has been reported as a heterozygous or compound heterozygous mutation in five Japanese patients (Yorifuji et al. 2011). The p.R933* mutation was reported in a patient who was compound heterozygous for the well‐studied c.3992‐9G>A splice site mutation (Thomas et al. 1995). Patient 1 suffered from severe hypoglycemia and was unresponsive to medical treatment; this patient underwent a pancreatectomy and exhibited focal histology, the plasma glucose concentrations remained normal now. The splicing mutation (c.1176+1G>A) detected in patient 9 was predicted to disrupt the acceptor splice site of intron 8; this mutation is different from the previously reported mutation (c.1176+2T>C) (Snider et al. 2013) but is closer to the splice site. Therefore, we hypothesized that the mutation could lead to CHI. Patient 16 was a compound heterozygote with frameshift mutations (p.Q953Rfs*89 and p.V1429Sfs*31), which were inherited from his father and mother respectively, his family refused to perform pancreatectomy and died after discharged from our hospital. A reported point mutation (c.2860C>T, p.Q954*) results in the introduction of a termination codon at position 954. The SUR1 protein product encoded by this mutant allele would be predicted to lack NBF‐2 (Nestorowicz, et al., 1998). The two frameshift mutations p.Q953Rfs*89 and p.V1429Sfs*31 would result in the introduction of a termination codon at position 1042 and 1460 respectively, thus resulting in a truncated protein product of SUR1.

Three previously reported missense mutations (p.F27S, p.R74W, p.E1506K) (Huopio et al. 2000; Suchi et al. 2003; Stanley et al. 2004) in *ABCC8* were identified. These mutations are in highly conserved regions. There is still controversy regarding the association between the p.E1506K mutation and diabetes type II (Pinney, et al., 2008; Vieira et al. 2010). The history of the families of our proband carrying the p.E1506K mutation does not agree with the theory that this mutation predisposes one to early‐onset type II diabetes. Patient 10 was compound heterozygous for the c.1279‐4A>G mutation in *GLUD1*, and her blood ammonia level was 147 *μ*mol/L (normal range: 11–35 *μ*mol/L). The mutation prediction analysis using web tools revealed a polymorphism. However, this variant is defined as a rare SNP in the 1000 Genomes Project Database (MAF = 0.0002), and it was absent in the 50 normal controls. The real function of this mutation may require further study.

Six novel mutations were found in *ABCC8*/*KCNJ11* in eight patients, and there were four patients carrying the same two mutations. The splicing mutation (c.2557‐1G>C) has not been previously reported; it is predicted to disrupt the acceptor splice site of intron 22. Interestingly, we found two mutations (*ABCC8*: p.T1042Qfs*75; *KCNJ11*: p.S103Pfs*28) in two patients. The indel mutation (c.3124‐3126delACCinsCAGCCAGGAACTG) causes a frameshift and introduces a premature stop codon 75 codons downstream of this mutation, thus leading to the loss of the functional domain NBD2, which appears to be the site for MgADP binding (Conti et al. 2001). MgADP and diazoxide activate a *β*‐ATP‐sensitive potassium channel in the presence of inhibitory concentrations of ATP, and both processes require Mg^2+^ (Shyng et al. 1997). Mutations in NBD2 can abolish channel activation by diazoxide or MgADP (Shyng et al. 1997). One reported phenotype associated with a dominant inherited mutation (p.G1479A) confirms the reported phenotype associated with dominant mutations in the NBD2 region (Pinney, et al., 2008). The mutational insertion of one nucleotide (c.305_306insG) causes premature termination 28 amino acids downstream. This mutation is located in the transmembrane domain 0 (TMD0) of SUR1. Previous studies have shown that the TMD0 domain of SUR1 mediates the strong association between SUR1 and Kir6.2, which modulates the trafficking and gating of the KATP channel (Chan et al. 2003).

A novel 55 nucleotide deletion was detected in patient 8 that spans from the end of the coding region to the noncoding region; this may result in a 16 amino acid deletion at the C‐terminus of SUR1. Because the C‐terminus of SUR1 contains an anterograde signal that is required for KATP channels to exit the ER/cis‐Golgi compartment and transit to the cell surface, a deletion of as few as the last seven amino acids from SUR1 markedly reduces the surface expression of KATP channels (Sharma et al. 1999).

A novel missense mutation (p.I49N) in *KCNJ11* was detected. The web‐based mutation prediction analysis revealed that this mutation is disease causing; this mutation is in a highly conserved region, and the patient was responsive to diazoxide treatment. Although synonymous mutations are typically assumed to have no effect, recent studies have demonstrated that certain nucleotide substitutions may affect mRNA splicing sites by inactivating an exonic splicing enhancer (ESE) (Cartegni et al. 2003). We used the ESEfinder web‐based resource to identify putative ESEs in a novel synonymous mutation (p.L281L) in patient 16. The finial values were below the threshold values (data not shown). Experimental evidence is necessary to clarify the implications for CHI.

Regarding other gene mutations, a novel deletion (c.37_39delAAG) was detected in *GCK* that results in a lysine deletion at position 13 in the amino acid sequence. This position is located close to the N‐terminus and is not part of any functional domain. Thus, we reasoned that this mutation is not the disease‐causing mutation in this patient.

*HADH* mutations are a rare cause of recessively inherited congenital hyperinsulinism. Recessive mutations in this gene were first identified in patients with specific fatty acid oxidation defects in which urinary 3‐hydroxyglutarate was present, and plasma 3‐hydroxybutyryl‐carnitine levels were elevated (Molven et al. 2004). However, a patient with homozygous *HADH* mutations but normal acylcarnitines and urine organic acids has been reported (Di Candia et al. 2009). A novel compound heterozygous mutation (p.R10P, p.V30E) was detected in patient 19; these missense mutations were predicted to be disease causing, and they are in highly conserved positions. The patient's acylcarnitines and urine organic acids were normal. Patient 1 also harbored a p.T240M mutation in *HADH*, which the web tool analysis revealed to be a polymorphism; this mutation is in a poorly conserved position, and this mutation was therefore assumed to be benign.

Mutations in *HNF4A* associated with CHI and noninsulin‐dependent diabetes have been reported (Sakurai et al. 2000; Stanescu et al. 2012). These mutations are inherited as a dominant form and are responsive to therapy for hypoglycemic hyperinsulinism. One previously reported (Sakurai et al. 2000) missense mutation (p.T139I) was detected in patient 21; this patient was diagnosed with NIDDM after 25 years of age, but a newborn with diazoxide‐responsive hyperinsulinism was found to have a known MODY1 mutation in *HNF4A* (Stanescu et al. 2012). These data demonstrate that mutations in *HNF4A* can cause hyperinsulinism early in life and diabetes later in life. For these patients with *HNF4A* mutations, a genetic diagnosis is important because this can be successfully managed with low‐dose sulfonylureas.

In conclusion, we detected mutations in 21 CHI patients, for a detection rate of approximately 65.6%, of the 21 patients, 19 could be diagnosed at the genomic level. Etiological diagnosis using genetics is an objective diagnostic method; an accurate and timely prediction of the phenotype based on the genotype is crucial for limiting the exposure to persistent hypoglycemia and reducing the risk of seizures and permanent brain damage in infants and children with CHI. We hope that the utilization of sequence analyses of CHI‐related genes will contribute to a better diagnosis and prognosis of Chinese patients with CHI.

## Conflict of Interest

The authors declare no conflicts of interest.
